# A Light-Weight Artificial Neural Network for Recognition of Activities of Daily Living

**DOI:** 10.3390/s23135854

**Published:** 2023-06-24

**Authors:** Samer A. Mohamed, Uriel Martinez-Hernandez

**Affiliations:** 1 Department of Electronic and Electrical Engineering, Faculty of Engineering and Design, University of Bath, Bath BA2 7AY, UK; 2 Mechatronics Engineering Department, Faculty of Engineering, Ain Shams University, Cairo 11566, Egypt; 3 Multimodal Inte-R-Action Lab, University of Bath, Bath BA2 7AY, UK

**Keywords:** deep learning, activity recognition, lower-limb motion recognition, wearable sensors

## Abstract

Human activity recognition (HAR) is essential for the development of robots to assist humans in daily activities. HAR is required to be accurate, fast and suitable for low-cost wearable devices to ensure portable and safe assistance. Current computational methods can achieve accurate recognition results but tend to be computationally expensive, making them unsuitable for the development of wearable robots in terms of speed and processing power. This paper proposes a light-weight architecture for recognition of activities using five inertial measurement units and four goniometers attached to the lower limb. First, a systematic extraction of time-domain features from wearable sensor data is performed. Second, a small high-speed artificial neural network and line search method for cost function optimization are used for activity recognition. The proposed method is systematically validated using a large dataset composed of wearable sensor data from seven activities (*sitting, standing, walking, stair ascent/descent, ramp ascent/descent*) associated with eight healthy subjects. The accuracy and speed results are compared against methods commonly used for activity recognition including deep neural networks, convolutional neural networks, long short-term memory and convolutional–long short-term memory hybrid networks. The experiments demonstrate that the light-weight architecture can achieve a high recognition accuracy of 98.60%, 93.10% and 84.77% for seen data from seen subjects, unseen data from seen subjects and unseen data from unseen subjects, respectively, and an inference time of 85 μs. The results show that the proposed approach can perform accurate and fast activity recognition with a reduced computational complexity suitable for the development of portable assistive devices.

## 1. Introduction

Locomotion is the ability to move from one place to another to perform activities of daily living independently [1]. This ability, which involves carrying out activities such as walking and stair and ramp ascent/descent, is commonly affected by aging and strokes, reducing the quality of life of individuals [2]. Recent studies have indicated that two-thirds of stroke survivors with lower-limb impairments are unable to continue with their professional career plans [3]. Active orthoses offer intelligent wearable devices to assist patients in improving their locomotive performance and recovering their quality of life [4]. These wearable devices need to understand the user’s motion intent to deliver a safe, controlled and timely assistance. This process, known as human activity recognition (HAR), is commonly performed using wearable sensors and computational methods implemented in assistive robots [5,6]. Even though sensing technology and computational intelligence have shown rapid progress in recent years, HAR still faces various challenges including critical accuracy demands, since recognition errors could result in faulty assistance. Moreover, real-time constraints of the associated control systems demand a high-speed inference, which is further challenged by the scarce computational resources of portable low-weight devices [7].

The goal for HAR is to maintain an accurate and fast intent prediction using portable low-weight devices and low-cost sensors. Sensor-based HAR is commonly classified into body-worn, object and ambient recognition. Object sensors are placed inside objects to measure their kinematic states, for example, accelerometers in mobile phones detect activities such as walking and running [8]. Ambient sensors, such as stereo cameras, capture changes in the environment to estimate what the subject might be doing in the perimeter [9]. Body-worn or wearable sensors are attached to the body at specific joints or links. Wearable sensors are widely adopted for HAR, since they convey rich features with sampling rates on the order of hundreds of hertz [10]. These sensors are highly portable compared to ambient and object sensors, making them suitable for indoor and outdoor activities. Wearable sensors include goniometers, inertial measurement units (IMUs) and electromyography (EMG) systems [11,12]. IMUs and goniometers offer an advantage for portable devices since they are light-weight and low-cost sensors [13]. The presented approach suggests attaching IMUs to the lower limb to track the angular velocity about the corresponding joint and the acceleration of the center point of that link. Goniometers attached to joints help track the angular trajectories contributing to the activity of interest. Time- and/or frequency-domain features can be extracted from these kinematic trajectories with negligible computational overhead, which eliminates the need for less robust sensor arrays such as EMG ones [14].

Wearable sensor data need preprocessing to eliminate anomalies and undesired noise [15]. The proposed preprocessing stage starts with data cleaning by discarding faulty motion circuits with missing entries or data. Motion axes are also assessed to discard irrelevant sensory channels with low-magnitude temporal variation. The labeled dataset is segmented into windows with small shifts in a one-dimensional convolutional fashion to group different samples of a parametric probability distribution [16]. The optimal window and shift sizes are selected based on a compromise between inference time and captured temporal pattern span. The proposed method emphasizes the importance of systematic feature extraction by defining relevant time-domain metrics [17]. On the one hand, this approach has a major advantage over stochastic feature extraction methods, which use empirical rules for hyperparameter tuning resulting in overly sized feature extractors [18]. On the other hand, systematic feature extraction using time/frequency-domain analysis produces powerful features for periodic lower-limb patterns, reducing the computational complexity of subsequent processes.

Verification methods are crucial for measuring the performance of machine learning classifiers [19,20]. It is necessary to ensure the desired properties of a complex system by evaluating statistical metrics. The most widely used curves for AI classifiers are learning curves, which display the evolution of classification accuracy during training for both seen and unseen data. Seen data refer to the portion of the dataset which is used to minimize the system’s cost function and reduce classification error. Unseen data refer to another portion of the dataset which measures the classifier’s performance on validation or testing data not previously seen by the network, to assess the classifier’s success on a wider range of the population. Confusion matrices are used to spot the classifier’s confusion patterns and how some classes could be systematically confused for others. This helps the designer tune the classifier’s performance by changing hyperparameter values, cost function or network structure to eliminate this confusion. A statistical analysis of classifier performance often also includes the F1-score to measure testing/validation accuracy. The F1-score is the harmonic mean of two other statistical measures known as precision and recall. The precision is the number of true positive results divided by the sum of all positive results, while the recall is the number of true positive results divided by the sum of all samples that should have been identified as positive.

This work presents a light-weight artificial neural network (ANN) architecture that uses feature windows with consecutive time stamps. This concept takes inspiration from recurrent neural networks, which use sequence modeling to combine past and present knowledge [21]. The proposed light-weight ANN method is trained to classify seven locomotion activities (*sitting*, *standing*, *level ground walking*, *ramp ascent* and *ramp descent*, *stair ascent* and *stair descent*) using the open-source ENABL3S dataset [22]. The classifier is systematically tested against deep learning techniques (deep neural network (DNN), convolutional neural network (CNN), long short-term memory (LSTM) and a CNN-LSTM hybrid) whose hyperparameters are carefully tuned using grid search to maximize competitiveness [23]. Eight K-fold validation experiments are carried out to assess the consistency of the proposed approach and deep learning techniques as well as their respective accuracy levels, F1-scores and average inference times. The light-weight ANN proves to be competitive, making it suitable for the development of portable assistive systems capable of recognizing activities in real time.

This paper is organized as follows: the related work is presented in Section 2. The proposed feature extraction3 and recognition methods along with the competing deep learning techniques are described in Section 3. The experiments and results are presented in Section 4. Section 5 and Section 6 present the discussion and conclusions, respectively.

## 2. Related Work

This section presents related works in the field of robotic orthoses and activity recognition. These works are compared in terms of hardware complexity, efficiency of computational methods and classification accuracy.

An exoskeleton or wearable orthotic device is a term that refers to any active method used to provide partial or full assistance to the muscular activity of the wearer. A wearable assistive device is a fully closed-loop system that starts with motion intent prediction and ends with controlled mechanical assistance. Lower-limb exoskeletons are classified into systems for multiple-joint actuation and for single-joint actuation [24]. Trunk–hip–knee–ankle–foot systems are the most complex since they span multiple degrees of freedom and are generally used to offer more stability in the trunk and hip. Other variations of multiple-joint systems can be realized by discarding a degree of freedom at a time such as: hip–knee–ankle–foot devices for flexion/extension and abduction/adduction control with free or locking motion in the hip joint, hip–knee devices for flexion/extension movements of hip and knee joints and knee–ankle–foot devices. Kao et al. [25] collected lower-body kinematic trajectories, EMG signals and ground reaction forces to compare gait patterns before and after an ankle–foot orthosis attachment. The study showed the importance of optimizing sensor modalities to match the required level of assistance and actuator response time. This optimization process is also dependent on the number of useful features that parameterize the data collected from several individuals. Device actuation is governed by important design considerations other than sensor modality optimization such as mechanical efficiency, size, weight and portability. Conventional actuators used in exoskeletons are electric actuators, pneumatic actuators, hydraulic actuators in addition to some modern actuators such as series elastic actuators and pneumatic artificial muscles [26].

Sensor data type plays a key role in HAR for reliable and safe assistive devices. IMUs, comprising accelerometer, gyroscope and magnetometer signals, are the most widely used wearable sensors in HAR. In particular, accelerometer and gyroscope signals measuring gravitational accelerations (*x*, *y* and *z* axes) and angular velocity (roll, yaw and pitch), respectively, offer relevant data, compared to magnetometer signals, for the design of HAR methods. Sampling rate adjustment is an important calibration step, since higher rates provide more accurate, precise feedback at the cost of power consumption and battery drain. Typical IMU sampling rates are on the order of several hundred to several thousand Hz. Electromyography (EMG) sensors are used in HAR given their capability to measure electrical signals from muscle contractions while performing activities. There are two EMG sensor types: surface EMG (sEMG) sensors, which are noninvasive electrodes placed on the skin surface [27] and intramuscular EMG (iEMG), which uses invasive elements embedded beneath the skin [28]. Other sensors used in HAR include mechanomyography, which measures low-frequency muscular contractions using accelerometers or microphones [29], electroencephalography for brain activity monitoring [30] and piezoelectric sensors that convert pressure loads to electrical signals [31]. HAR is not limited to these sensors; however, the above-mentioned devices are the most widely used in academia and the industry. The selection of sensory channels is dependent on the number of recognized activities and their features. A large number of classes requires sensory information that is rich enough to convey a suitable number of useful features.

The design of computational methods for activity recognition has been widely investigated with a variety of approaches ranging from heuristic methods to neural network approaches and advanced deep learning techniques. Some works propose simple unsupervised approaches such as k-nearest neighbors as in [32] to predict six different activities based on a single smartphone IMU data including laying, downstairs walking, sitting, upstairs walking, standing and walking. The formerly mentioned k-nearest neighbors approach yields a maximum validation accuracy of 90.46% and is not able to recognize some dynamic activities such as ramp ascent/descent. Support vector machines also belong to the same category of computationally efficient methods and have been employed by Tran et al. to classify the previously mentioned six activities using a smart-phone IMU [33]. The group passed 248 useful features to the support vector machine but only managed to achieve a maximum validation accuracy of 89.59%. ANNs have paved the way for more intricate classification methods starting with small neural networks that can achieve acceptable results with low-cost microcomputing devices. Jmal et al. [34] deployed a high-speed light ANN on a microcomputer for recognizing three activities (sitting, walking and running). The approach proved to be computationally efficient but achieved an overall maximum accuracy of 86% with a single IMU attached to the ankle. Such conventional approaches are computationally cheap but require the systematic handcrafting of features to produce accurate classification results.

Recent works have explored the potential of end-to-end deep learning methods for activity recognition using raw input data from a variety of sensors, e.g., IMUs and EMG sensors. CNNs have proved to be highly efficient in automatic feature extraction for image processing applications, and the same principle has been explored in HAR using the wearable sensor data in an imagelike format [35]. While CNNs have some remarkable advantages such as parameter sharing and a sparsity of connections, they require higher computational effort than conventional approaches such as k-nearest neighbors, support vector machines and single-layer ANNs. Male et al. used an LSTM network to merge data from IMU and vision sensors for activity recognition [36]. That approach achieved accurate results; however, the vision component limited the method to fixed and well-controlled environments. Some recent works have emphasized the importance of systematic feature engineering for machine learning and deep learning methods to improve classification accuracy [37]. Wang et al. used genetic algorithms to select relevant sensory inputs and a Bayesian approach for deep CNN hyperparameter tuning, achieving a 90% activity recognition accuracy for unseen subjects using feedback from 24 sensory channels [38]. Recurrent neural networks use previous outputs as current inputs while maintaining a hidden state, which is useful for HAR research since the input is a time series. Ghislieri et al. proposed a binary classification LSTM network for muscle activity detection, achieving an average validation accuracy of 92% [39]. A multilevel classifier based on a CNN-LSTM hybrid proposed in [40] combined the feature extraction capability of CNNs and the state evolution tracking from recurrent neural networks, achieving a mean F1-score of 0.97 and a validation accuracy of 94.53% for ten healthy subjects using two IMUs. However, the training dataset for that approach was not balanced and a class bias was expected. Wang et al. proposed a similar hybrid approach [41] achieving a 95.87% validation accuracy on the recognition of six activities (walking, lying, sitting, standing, stair ascent and stair descent) using a smart-phone IMU. Despite the high validation accuracy of the approach, it ignored some dynamic activities such as ramp ascent/descent and was very computationally demanding. Another CNN-LSTM hybrid approach was developed by Jain et al. to classify six activities (standing, sitting, lying, level walking, walking downstairs and walking upstairs) and transitions between states, achieving an average F1-score of 0.8782 [42].

This paper proposes a light-weight computational architecture exploiting the benefits of combining feature engineering and artificial neural networks. This approach uses wearable sensor data for the classification of seven activities including *sitting*, *standing*, *ground level walking*, *ramp ascent* and *ramp descent*, *stair ascent* and *stair descent*. The proposed approach uses sensor data available in the ENABL3S benchmark dataset [22]. The key idea for the proposed light-weight ANN architecture is to achieve a competitive performance compared to deep learning approaches, in terms of classification accuracy and F1-score, while maintaining smaller training and inference times and computational complexity through the systematic extraction of features.

## 3. Methods

**Figure 1:**
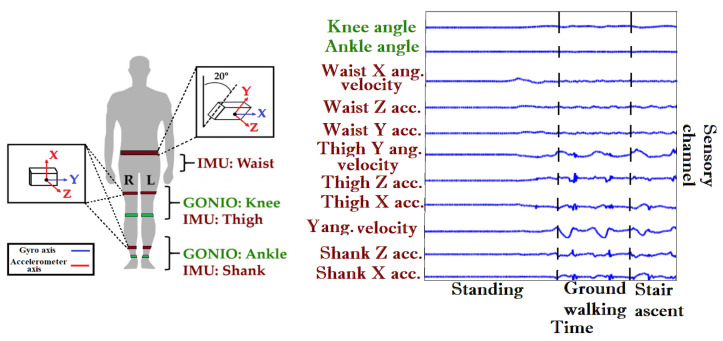
Hardware setup and sensory channels, where green and red colors represent goniometers and inertial measurement units, respectively (figure adapted from [22]).

This section describes the light-weight ANN network approach starting with input preprocessing, hyperparameter tuning and ending with the cost function optimization along with the network training procedure. This work also implemented a set of methods for benchmarking including DNNs, CNNs, LSTM and CNN-LSTM hybrid networks. The ENABL3S dataset [22] used in this research work comprises raw sensory information from a set of sensors synchronized at a constant refresh rate of 500 Hz. In order to optimize sensor modalities, only relevant sensory channels were selected from the dataset. The sensors of interest for this study were five six-DOF IMUs (MPU-9250; Invensense, San Jose, CA, USA) attached to the waist and both thighs and shanks of test subjects (tilted 20 deg from vertical), and four goniometers (SG150; Biometrics Ltd., Newport, UK) attached to both knees and ankles. The sensor type, attachment location and sensory channels used for feature extraction are illustrated in Figure 1. The sensory channels were selected based on their relevance to forward motion, where only the forward acceleration of movable links as well as their associated angular displacements and velocities were considered. In total, the data used were composed of accelerometer raw readings (*x* and *y* axes) [43] and angular velocity (*x* axis) from the waist, acceleration (*x* and *z* axes) from the thigh and shank, angular velocity (*y* axis) from the thigh and shank and absolute angle per goniometer from ankle and knee. Benchmarking is implemented with eight k-fold experiments per computational method. For each fold, data from seven individuals were used for training and validation (seen subjects), and data from the eighth excluded subject were used for testing (unseen subject). The training dataset for each fold comprised data points from 56 motion circuits (8 seen circuits per training seen subject), the validation dataset comprised 14 motion circuits (2 unseen circuits per seen subject), and the test dataset comprised 4 unseen motion circuits from the unseen test subject.

A total of 74 motion circuits for eight healthy subjects with different biometrics were selected from the ENABL3S dataset as shown in Table 1. Even-numbered circuits recorded the following sequence of activities: *sitting* → *standing* → *level walking* → *ramp ascent* → *level walking* → *stair descent* → *level walking* → *standing* → *sitting*. Odd-numbered circuits recorded activities in a reverse order: *sitting* → *standing* → *level walking* → *stair ascent* → *level walking* → *ramp descent* → *level walking* → *standing* → *sitting*. The eight folds were used to assess the consistency of the results for different data distributions. The results assessed the performance metrics in terms of the compromise between classification accuracy, computational cost and speed. The motion circuits were preprocessed through windowing, shifting, manual feature extraction or a combination of the three, yielding eight segmented datasets per method. For each fold, the learning curves, testing accuracy, confusion matrices, F1-scores and average inference times were measured for training, validation and testing subsets to assess the performance on seen and unseen data from seen and unseen subjects. The F1-score was computed using the following equation:

(1) $$F1=\frac{TP}{TP+\frac{1}{2}\left(FP+FN\right)}$$

where TP, FP and FN are the true positive, false positive and false negative results, respectively. Two further training trials were conducted to validate the optimal selection of sensory channels for the proposed approach. The two trials investigated the performance of the network on seen subjects by using only IMUs for the first trial and only goniometers for the second.

### 3.1. Shallow Neural Network

#### 3.1.1. ANN Preprocessing

**Figure 2:**
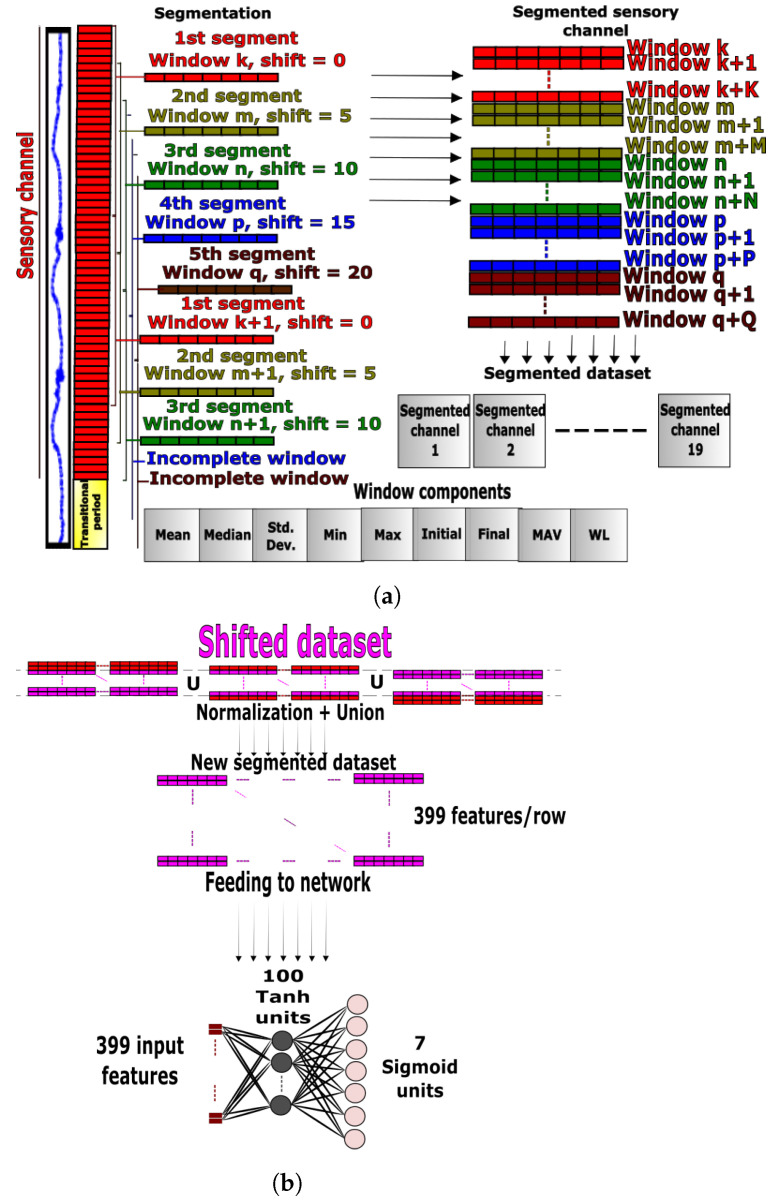
Data preprocessing and ANN architecture. (**a**) Grouping readings into time-domain feature windows. (**b**) Union of past and present feature windows for ANN.

The data preprocessing stage started with channel segmentation, where each sensory channel was divided into fixed-size windows. The dataset readings were sampled at a rate of 500 Hz, therefore a window size of twenty-five samples was selected to capture enough features without exceeding a time threshold (around 0.05 s). A transitional region of five hundred samples (1 s) between two successive activities was removed to account for the confusion caused by transitional periods (e.g., swift transition between *ground-level walking* and *standing*). This ensured that correct ground-truth labels were included in the dataset. A window shift of five samples was introduced for each new segmentation session causing a time shift of about 0.01 s between successive segments. The time shift was applied five times to span the whole range and produce a rich dataset. Different combinations of window size and shifts were applied to select the optimal segmentation values using a grid search (e.g., 25 samples per window, 5 samples per window shift, and 500 samples removal per transitional period). Known time- and frequency-domain features were investigated, and training trials were conducted using different combinations of features, based on which, the optimal set of features was selected. For each window, the following nine features were extracted: (1) mean, (2) median, (3) standard deviation, (4) minimum, (5) maximum, (6) initial value, (7) final value, (8) mean absolute value (MAV) and (9) waveform length (WL), as follows:

(2) $$\mu^{w}=\frac{1}{25}\sum_{n=i}^{i+24}x_{n}^{w}$$

(3) $${\tilde{M}}^{w}=x_{i+12}^{w}$$

(4) $$\sigma^{w}=\sqrt{\frac{\sum_{n=i}^{i+24}{\left(x_{n}^{w}-\mu^{w}\right)}^{2}}{25}}$$

(5) $$Min^{w}=\arg\min_{x_{n}^{w}}$$

(6) $$Max^{w}=\arg\max_{x_{n}^{w}}$$

(7) $$I^{w}=x_{i}^{w}$$

(8) $$F^{w}=x_{i+24}^{w}$$

(9) $$MAV^{w}=\frac{\sum_{n=i}^{i+24}|x_{n}^{w}-\mu^{w}|}{25}$$

(10) $$WL^{w}=\sum_{n=i}^{i+23}|x_{n+1}^{w}-x_{n}^{w}|$$

where *i*, $\mu^{w}$, ${\tilde{M}}^{w}$, $\sigma^{w}$, $MAV^{w}$ and $WL^{w}$ represent the index of the first element of the window, mean, median, standard deviation, mean absolute value and waveform length, respectively, for the window *w*. The parameters $x_{n}^{w}$, $Min^{w}$, $Max^{w}$, $I^{w}$ and $F^{w}$ represent the minimum, maximum, initial and final readings, respectively, for the window *w*. Incomplete windows or residuals were removed to standardize the amount of captured variation per window, and each fully segmented sensory channel was appended to the final preprocessed dataset (see Figure 2a). Mean normalization and feature scaling were applied to the processed data samples before feeding. Subsequently, a downsampling technique was used to balance the labels and avoid class bias. Downsampling reduces the number of class labels to matching numbers; this technique was selected because it helped reduce the quantity of redundant information of periodic repetitive data collected from gait activities.

#### 3.1.2. ANN Design

For sequential data, information from past samples has the potential to improve the classification accuracy. The proposed approach captured this by embedding data from past windows within input features. Three consecutive windows were concatenated and fed to the classifier as a whole, instead of feeding individual windows. First, the segmented dataset was copied three times, where each copy was shifted one window below its predecessor. The three copies were then concatenated to form a new dataset. This approach was simple and highly effective for activity recognition, since it considered past and current inputs. This functionality in the proposed light-weight ANN approach resembles that of an RNN, except that the ANN gradient computation and training are considerably faster. The classifier hyperparameters, including the number of past windows, were tuned experimentally using a grid search yielding a network with a single hidden layer and 100 hidden units. For the activation on hidden neurons, the Tanh function was employed, while a Sigmoid function was used for the activation of the output neurons composed of seven units corresponding to the number of ADLs (see Figure 2b).

#### 3.1.3. ANN Training

The dataset labels were converted to a one-hot representation and the optimization function fmincg [44] minimized the cross-entropy objective function of one training batch for 1000 epochs using a line search algorithm. Line search provides a better rate of convergence for the ANN cost function than traditional gradient descent at a higher computational cost. The algorithm uses the Wolfe conditions to optimize the step size without manual tuning of a fixed learning rate [45]. Line search achieved better training results for the proposed ANN than gradient descent variants such as RMSProp and Adam algorithms without any parameter tuning. L2 regularization was applied to prevent overfitting with a regularization parameter equal to 2. The regularization parameter was tuned to reduce the error between training and validation accuracy. The simplicity of the architecture justified the usage of the computationally demanding line search algorithm, embedded within the fmincg function, given the small effort required for gradient computing of the small ANN.

### 3.2. Deep Neural Network

#### 3.2.1. DNN Preprocessing

**Figure 3:**
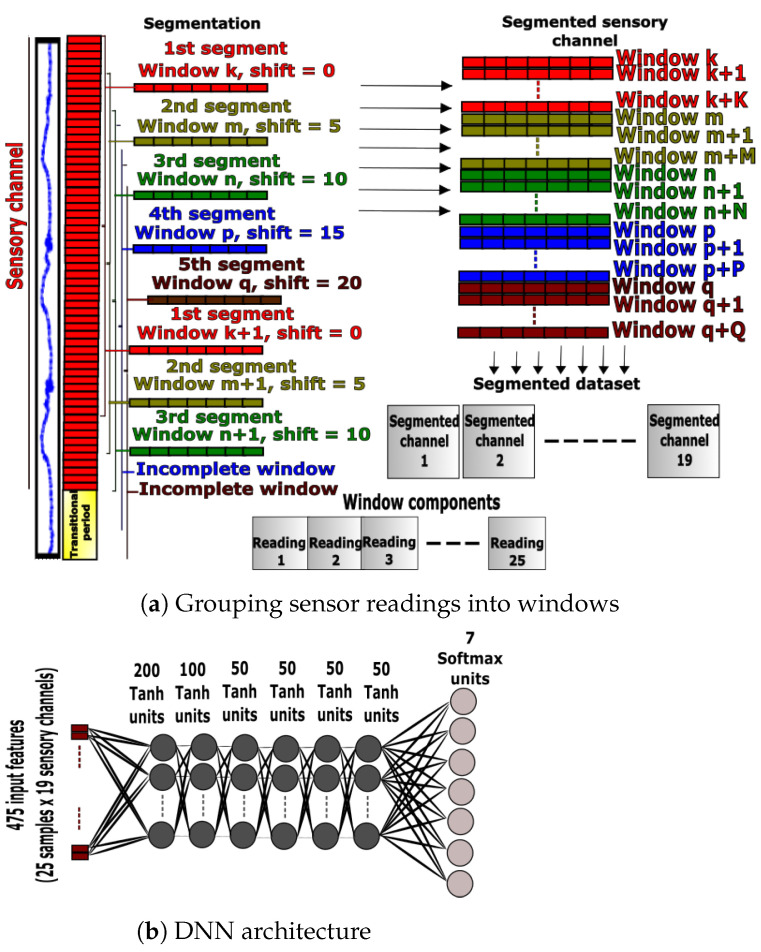
Data preprocessing and DNN network architecture.

The sensory channels shown in Figure 1 were used for activity recognition with the DNN approach. This deep learning approach used the preprocessing steps employed by the proposed ANN method in Section 3.1.1. These preprocessing steps started with data segmentation into five different segments, each of the five samples ahead of its predecessor. No handcrafted features were computed for the windows; instead, the raw signals composed of 25 samples were directly stacked into windows for input to the DNN. Transitional periods and incomplete windows were removed and finally, segmented channels were appended to the segmented dataset as shown in Figure 3a. Mean normalization and feature scaling processes were applied to the dataset before feeding. The downsampling process was also used to balance the classes.

#### 3.2.2. DNN Design and Training

Multilayer networks are used for the classification of complex patterns, since the first layers act as feature extractors and the final layer performs classification tasks [46]. Therefore, a manual extraction of features is eliminated in favor of a more stochastic approach towards feature extraction. The hyperparameters of the classifier including the number of layers, hidden units, regularization and activation functions were tuned using a grid search to maximize the validation accuracy and reduce overfitting. The final network had six hidden layers with a Tanh activation function and L2 regularization, followed by a final Softmax layer corresponding to different activities, as shown in Figure 3b. The Adadelta optimizer with a 0.05 learning rate was used to minimize the cross-entropy cost function of the training batch for 10,000 epochs.

### 3.3. Convolutional Neural Network

#### 3.3.1. CNN Preprocessing

**Figure 4:**
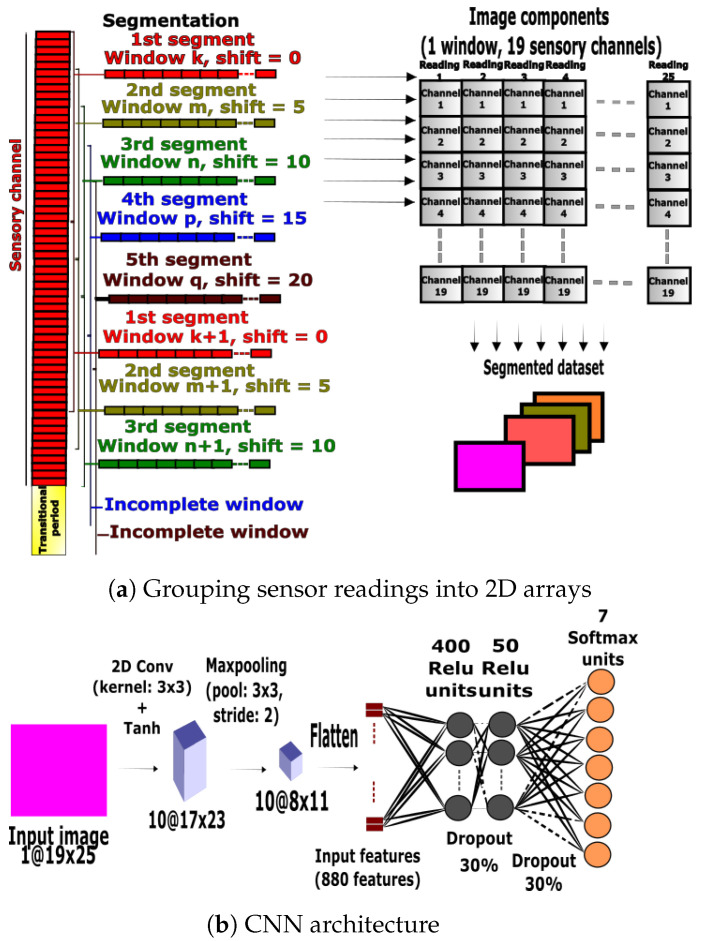
Data preprocessing and network architecture for the CNN.

The second benchmarking approach implemented a CNN that used 2D convolutions for feature extraction followed by an output classification layer. The same nineteen IMU and goniometer channels used for the light-weight ANN and DNN approaches were selected and preprocessed. The data were split into five segments, each comprised of 25-sample windows with 5-sample shifts. Transitional periods and incomplete windows were removed, and the raw feature windows were stacked together to form 2D arrays fed to the CNN. The rows and columns of the array represented the number of sensory channels and data samples. The 2D arrays were fed directly to the CNN as illustrated in Figure 4a. The classes were balanced using the downsampling technique after the preprocessing steps.

#### 3.3.2. CNN Design and Training

The CNN used a 2D convolutional layer composed of ten 3×3 kernels with a Tanh activation function, followed by a maxpooling layer of size 3×3, a 2-step stride and valid padding. Then, the layer was flattened and fed to two fully connected layers with a 30% dropout probability. The first and second layers had 400 and 500 hidden units, respectively, with a Relu activation. The final activations were fed to a Softmax layer (see Figure 4b). The categorical cross-entropy function was optimized using the Adadelta optimizer for a single full batch with a 0.05 learning rate for 4000 epochs.

### 3.4. Long Short-Term Network

#### 3.4.1. LSTM Preprocessing

**Figure 5:**
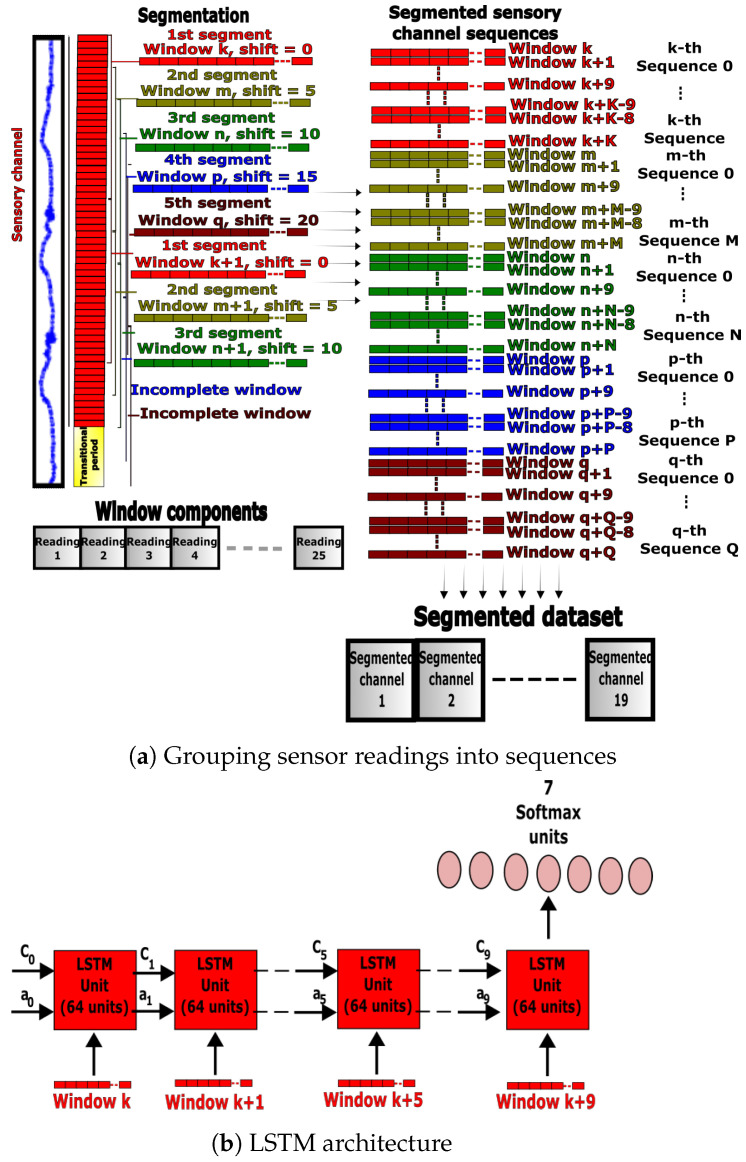
Data preprocessing and LSTM network architecture.

The third benchmarking approach used an LSTM network following the approach proposed in [47,48]. LSTM networks are known for their ability to process sequences of inputs instead of processing a single input at a time, which can enhance the classification accuracy. Data from the nineteen sensory channels were split into five segments, each comprised of 25-sample windows with 5-sample shifts. Moreover, each of the ten consecutive windows in a segment was bundled together to form a sequence of RNN inputs. Transitional periods and incomplete sequences were removed, and the data were downsampled to ensure balanced classes. A sequence of 10 windows without normalization was fed to the LSTM network at a time for the recognition of ADLs, as shown in Figure 5a.

#### 3.4.2. LSTM Design and Training

The hyperparameters of the classifier were tuned using a grid search to maximize the validation accuracy and optimize the computational effort. This process was applied to the number of layers, units and sequence size. The final network had one LSTM layer with 64 activation units, followed by a Softmax layer with 7 units corresponding to the activity classes (see Figure 5b). The initial hidden state and activation were both initialized to zeros. The Adadelta optimizer with a 0.05 learning rate was used to minimize the training batch cross-entropy cost function for 10 epochs.

### 3.5. CNN-LSTM Hybrid Network

#### 3.5.1. CNN-LSTM Preprocessing

**Figure 6:**
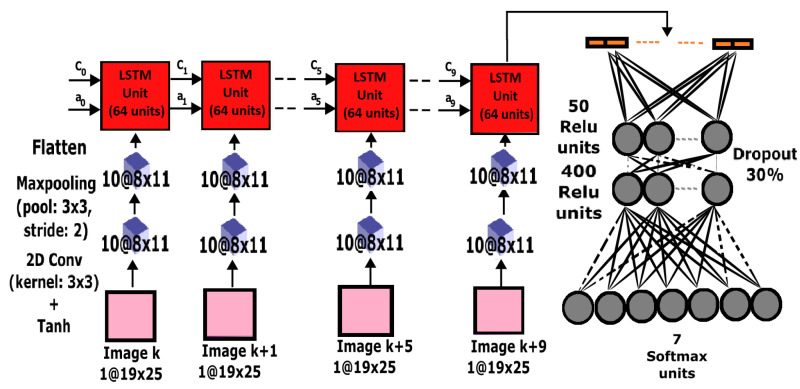
CNN-LSTM network architecture.

The combination of CNN feature extractors and LSTM sequence models can result in enhanced activity recognition as proposed in [40,49]. The preprocessing steps used for the CNN in Section 3.3.1 and Figure 6 were employed for the hybrid CNN-LSTM approach. In this hybrid approach, there was an additional final step in which ten consecutive 2D arrays in a segment were bundled together to form a sequence of LSTM inputs. Thus, 2D array sequences were used instead of window sequences. Each ten-array sequence was finally fed to the CNN-LSTM hybrid at a time. Similar to the previous approaches, the data were also downsampled to ensure balanced classes after the preprocessing stage.

#### 3.5.2. CNN-LSTM Design and Training

The CNN feature extractor consisted of a 2D convolutional layer with ten 3×3 kernels and a Tanh activation function. This layer was followed by a maxpooling layer of size 3×3 with a 2-step stride and valid padding. The layer was flattened and fed to an LSTM unit with 64 activation units. The CNN-LSTM output from the ten-array sequence was fed to two fully connected layers with a 30% dropout probability. The first layer had 400 hidden units while the second layer had 50 hidden units, both layers using Relu activation functions. The output from the fully connected layers was fed to a Softmax layer as shown in Figure 6. The categorical cross-entropy function was optimized using the Adadelta optimizer for a single full batch with a 0.05 learning rate for 20 epochs.

## 4. Results

In this section, the results obtained by training, validating and testing the networks are presented. The preprocessing methods introduced in the methods section were used to obtain useful features, the features were fed to the networks, and the weights were iteratively updated to minimize the cost functions. Finally, the learning curves, confusion matrices and F1-scores were displayed.

### 4.1. Activity Recognition for Seen Subjects

Test subjects whose motion data were used to train a network were referred to as seen subjects. This section presents the ADL classification results using data collected from seven subjects distributed among eight folds.

#### 4.1.1. ANN Approach

**Figure 7:**
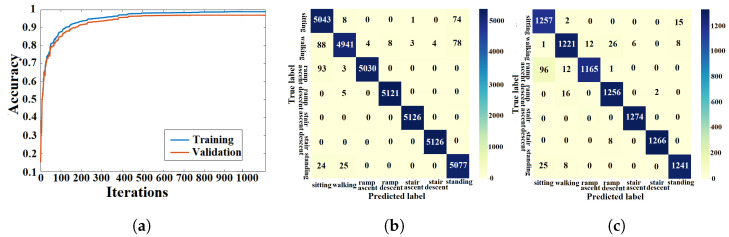
Training and validation results of the light-weight ANN with seen subjects. (**a**) ANN learning curve. (**b**) Training confusion matrix. (**c**) Validation confusion matrix.

The light-weight ANN cost function was optimized using the training algorithm described in Section 3.1.3. The training and validation processes of the ANN were performed using data from seen and unseen circuits, respectively. The learning curves from the k-fold experiment with the AB185 subject as the unseen subject are shown in Figure 7a. Confusion matrices from training and validation of the proposed ANN for the considered k-fold analysis are shown in Figure 7b and Figure 7c, respectively. The averaged training and validation accuracy results over the eight folds are shown in Table 2. The proposed ANN validation F1-scores for the seven classes (*sitting*, *standing*, *level walking*, *stair ascent*, *stair descent*, *ramp ascent* and *ramp descent*) were computed for the eight folds. The F1-scores for the eight folds were averaged (see Table 3). The light-weight ANN average input preprocessing duration was 38.75±342.1 μs and the average inference time was 45.79±619.05 μs, which yielded a total average run time of 84.54 μs for the pipeline.

#### 4.1.2. DNN Approach

**Figure 8:**
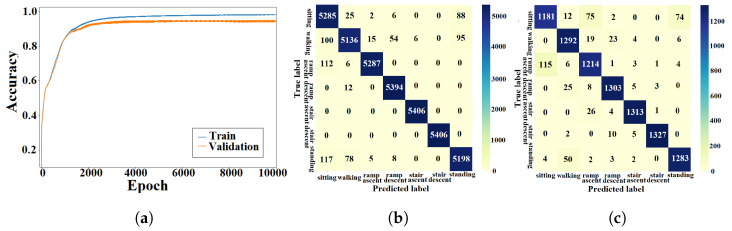
Training and validation results of the DNN with seen subjects. (**a**) DNN learning curve. (**b**) Training confusion matrix. (**c**) Validation confusion matrix.

The DNN cost function was optimized using the training algorithm described in Section 3.2.2. The DNN was trained and validated using data from seen and unseen circuits, respectively. The accuracy results for the k-fold experiment with the AB185 subject as the unseen subject are shown by learning curves in Figure 8a. Confusion matrices from the DNN training and validation are shown in Figure 8b,c. The average DNN validation accuracy over the eight folds is shown in Table 2. Training and validation F1-scores are shown in Table 3. The DNN average inference time was 3.09±0.33 ms.

#### 4.1.3. CNN Approach

**Figure 9:**
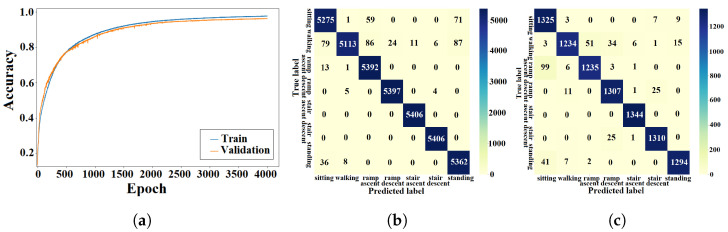
Training and validation results of the CNN with seen subjects. (**a**) CNN learning curve. (**b**) Training confusion matrix. (**c**) Validation confusion matrix.

The CNN design presented in Section 3.3.2 was trained using data from 56 seen circuits and validated using data from 14 unseen circuits. The learning curves for the k-fold experiment with the AB185 subject as the unseen subject are shown in Figure 9a. Training and validation results are shown via confusion matrices in Figure 9b,c. The average training and validation accuracy results for the eight folds are shown in Table 2. The validation F1-scores for the seven classes were computed and averaged over all eight folds. Table 3 shows the average F1-score values for the ANN and CNN. The CNN average inference time was 2.8±0.24 ms.

#### 4.1.4. LSTM Approach

**Figure 10:**
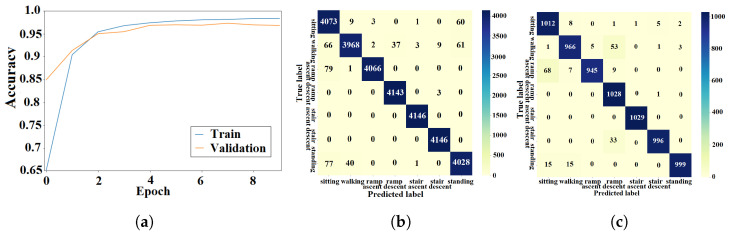
Training and validation results of the LSTM approach with seen subjects. (**a**) LSTM learning curve; (**b**) Training confusion matrix; (**c**) Validation confusion matrix.

The LSTM network was trained using the methods described in Section 3.4.2. The learning curves for the k-fold experiment treating the AB185 subject as the unseen subject are shown in Figure 10a. Confusion matrices from the training and validation processes of the LSTM are shown in Figure 10b and Figure 10c, respectively. The average k-fold training and validation accuracy results are presented in Table 2. Similarly, the averaged validation F1-scores for the seven classes can be found in Table 3. The LSTM average inference time was 5.86±0.97 ms.

#### 4.1.5. CNN-LSTM Approach

**Figure 11:**
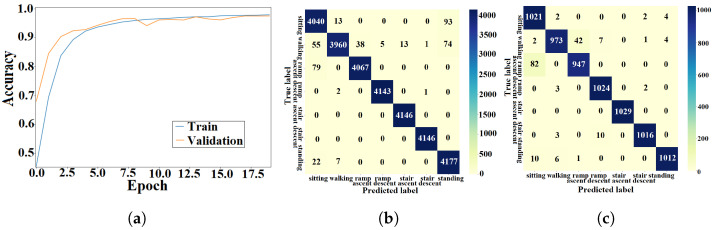
Training and validation results of the CNN-LSTM approach with seen subjects. (**a**) CNN-LSTM learning curve. (**b**) Training confusion matrix. (**c**) Validation confusion matrix.

The learning curves for the k-fold experiment using the AB185 subject as the unseen subject are shown in Figure 11a. The confusion matrices for training and validation are shown in Figure 11b,c. The averaged k-fold validation accuracy for the CNN-LSTM hybrid is shown in Table 2. Table 3 shows the average validation F1-scores for the seven classes computed for each fold. An average inference time of 6.57±0.9 ms was assigned to the CNN-LSTM hybrid approach.

#### 4.1.6. Accuracy of Light-Weight ANN on Seen Subjects with Individual and Combined Sensory Channels

The training and validation accuracy levels were validated for the proposed approach using the combined features of IMUs and goniometers. The validation accuracy level dropped by eliminating either IMUs or goniometers, which validated the originally proposed combination of IMU and goniometer channels as shown in Table 4. The validation F1-scores associated with each trial are shown in Table 5.

### 4.2. Activity Recognition for Unseen Subjects

This section presents the response of the ADL classification methods to unseen data from new unseen subjects. In these experiments, unseen subjects referred to test subjects whose motion circuit data were not used for network training and validation. The performance of each computational method on unseen subjects was assessed as in Section 4.1.

#### 4.2.1. ANN Approach

**Figure 12:**
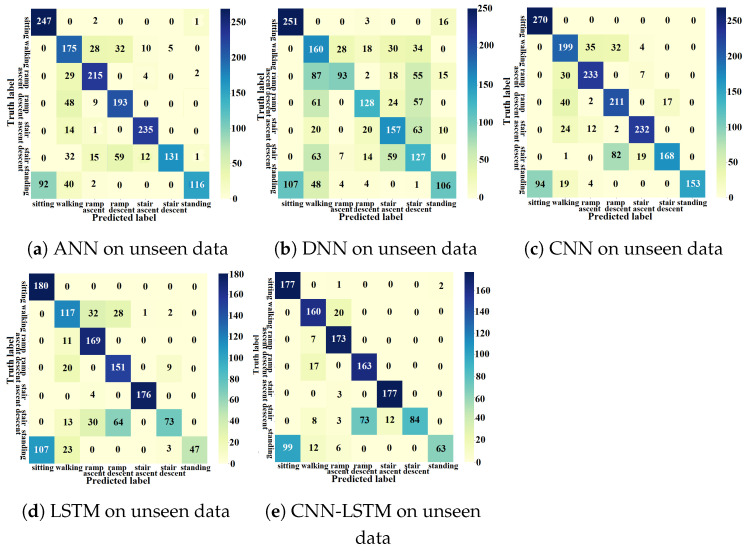
Unseen data’s confusion matrices.

The testing process of the proposed light-weight ANN used data from unseen motion circuits associated with unseen test subjects. The confusion matrix from the k-fold experiments using the AB185 subject as the unseen subject is shown in Figure 12a. The averaged accuracy results from the unseen-subject testing process are shown in Table 6. The ANN’s testing F1-scores from the seven activity classes, shown in Table 7, were computed for the unseen subject data for the eight folds and then averaged, with the lowest average F1-score being assigned to the *walking* class.

#### 4.2.2. DNN Approach

Unseen subjects’ data were used to test the DNN methods on activity recognition for unseen test subjects. Recognition accuracy for each activity is shown by the confusion matrix in Figure 12b, obtained from the k-fold experiment with the AB185 subject as the unseen subject. The average activity recognition accuracy with unseen subject data are shown in Table 6. The average F1-score results with unseen subject data for the eight folds are shown in Table 7. The lowest average F1-score was associated with the *ramp descent* class.

#### 4.2.3. CNN Approach

The CNN was tested using data from unseen test subjects. The confusion matrix in Figure 12c shows the recognition accuracy from the k-fold experiment with the AB185 subject as the unseen subject. The recognition accuracy results using unseen subjects for the eight folds were averaged as shown in Table 6. The testing F1-scores for unseen subject data and the eight folds were computed and averaged as shown in Table 7, where the lowest average F1-score was related to the *walking* class.

#### 4.2.4. LSTM Approach

The LSTM network for unseen-subject activity recognition was tested using data from the four testing motion circuits of the unseen test subject. The LSTM network testing confusion matrix for unseen data from the AB185 unseen subject is shown in Figure 12d. The averaged accuracy results over the eight folds are shown in Table 6. The testing F1-scores associated with the unseen subject data for the eight folds were computed and averaged as shown in Table 7. The lowest average F1-score was associated with the *walking* activity class.

#### 4.2.5. CNN-LSTM Approach

The CNN-LSTM testing (i.e., unseen circuits from the AB185 unseen subject) confusion matrix is shown in Figure 12e. The average testing accuracy over the eight folds and the average testing F1-scores for the seven classes are shown in Table 6 and Table 7, respectively.

#### 4.2.6. Accuracy of Light-Weight ANN on Unseen Subjects with Individual and Combined Sensors

The testing accuracy was validated for the light-weight ANN using the combined features of IMUs and goniometers. A series of two training trials was conducted for different sensor combinations to test the performance of the network on unseen subjects. The two trials showed that the average testing accuracy and F1-scores dropped by eliminating either IMUs or goniometers, which validated the originally proposed combination of IMU and goniometer channels as shown in Table 8 and Table 9.

## 5. Discussion

This section discusses the key differences between the proposed light-weight ANN method and the set of benchmarking approaches in terms of classification accuracy, inference speed and response to seen and unseen data.

### 5.1. Classification Accuracy

The training and validation learning curves showed a convergence toward a stable maximum accuracy level without intermittent spikes as shown in Figure 7a, Figure 8a, Figure 9a, Figure 10a and Figure 11a, which validated the hyperparameter tuning procedure. The proposed light-weight ANN method and the LSTM proposed in [39] had the highest average validation accuracy percentages of 93.102% and 92.99%, respectively. The light-weight ANN method showed a better performance on data from seen subjects compared to the other methods. This can be attributed to the systematic extraction of features that accurately parameterized the probabilistic distribution of the considered population, without relying on a stochastic feature extraction approach. This performance improvement can be also related to the use of the features extracted from multiple past windows to maintain a form of internal memory. The results from seen subjects showed that the proposed method was more powerful for relatively small datasets prepared for seen device users, which made it more convenient for the considered application. The ANN’s small training effort facilitated device tuning to accommodate a new user and eliminated the need for huge datasets or oversized computational resources. However, the CNN, LSTM and hybrid approaches had higher average accuracy percentages of 86.78%, 86.9% and 86.36% for data from unseen subjects compared to an accuracy of 84.77% for the light-weight ANN approach. The CNN-LSTM performance for activity recognition was tested using data from unseen test subjects. The averaged testing accuracy over the eight folds, shown in Table 6, indicated a slightly lower testing accuracy for the CNN-LSTM compared to LSTM and CNN approaches. This suggests that these deep learning methods are able to generalize to larger unseen populations, which makes them more convenient for training on very large datasets.

### 5.2. Inference Speed

The mean and variance of the inference speed for the presented computational methods were measured using 1000 samples each. A machine with an i7 Intel core and Nvidia RTX2060 graphics card was used to train, validate and test the approaches. MATLAB was used for the training and development of the light-weight ANN approach, while Tensorflow was used for the training, validation and testing of the rest of the approaches. The results showed a clear advantage in processing speed for the light-weight ANN approach over the other deep learning methods. The DNN average inference time was approximately 35 times greater than the average time required by the proposed light-weight ANN. The CNN average inference time of 2.8±0.24 ms was approximately 33 times greater than the average inference time required by the ANN. The LSTM average inference time was 5.86±0.97 ms, which is about 69 times greater than the inference time from the proposed ANN method. The average inference time of 6.57±0.9 ms for the CNN-LSTM hybrid was approximately 77 times higher than that of the proposed light-weight ANN method. The light-weight ANN achieved an inference speed that was at least 30 times faster than the fastest deep learning approach. This study used an offline dataset, which means that hardware restrictions were not considered. However, for a real-time scenario, the designer must account for hardware overhead. The designer must also embed a synchronization mechanism to feed sensory information to the network simultaneously without considerable lag. These aspects for the implementation in real time will be investigated in our future work. The average inference time for the proposed ANN method was 85 μs. The hyperparameters of the CNN method proposed in [35,36] were tuned using a grid search method to achieve the maximum classification accuracy with optimal network size and yet the inference time was 2.8 ms, which is 33 times higher than the mean inference time of the proposed ANN. The CNN-LSTM hybrid method proposed in [40,42] was adopted for benchmarking, and despite the high testing accuracy percentage for unseen subjects, the measured inference time was 6.57 ms, which is about 77 times higher than the one achieved by the light-weight ANN. Moreover, the deep learning methods relied on GPU utilities to achieve the measured speeds, while the light-weight ANN did not explicitly use GPUs. This shows that the proposed approach is optimal for embedded devices with low computational budgets. The fast inference achieved by the light-weight ANN approach can be related to the optimized size of the network, which consisted of one hidden layer with 100 hidden units. The systematic feature extraction method helped to extract useful features in approximately 46 μs on average without adopting additional costly computational layers based on stochastic techniques.

### 5.3. Confusion Patterns

The confusion matrices and F1-scores of the proposed approach and deep learning methods for benchmarking showed confusing trends. Confusion matrices from the DNN training and validation showed a larger proportion of off-diagonal elements compared to those of the proposed light-weight ANN (see Figure 8b,c. The average DNN validation F1-scores for the seven ADLs, shown in Table 3, were generally less than their light-weight ANN counterparts, except for the *stair ascent* class. The averaged validation F1-scores for the seven classes were approximately equal for the ANN and LSTM (see Table 3). A visual inspection of the confusion matrix of the ANN on unseen subjects indicated some confusing trends such as *standing* being heavily confused with *sitting* or *walking*, *stair descent* being confused with other activities, particularly *ramp descent* and *walking*, *ramp descent* being confused with *walking* and *walking* being confused with other states in general. The DNN recognition results on unseen subjects showed confusing trends such as *standing* being heavily confused with *sitting* or *walking* and *walking* being confused with other states. It was also shown that *stair descent* was confused multiple times with *stair ascent* or *walking*, *stair ascent* was likely to get confused with *stair descent*, *ramp descent* was heavily confused with *stair descent* or *walking*, and *ramp ascent* was heavily confused with *walking*. The results from the CNN on unseen subjects showed that *standing* was heavily confused with *sitting*, *stair descent* was heavily confused with *ramp descent*, *ramp descent* and *ramp ascent* were confused with *walking* and vice versa. The recognition outputs of the *sitting* class were all classified as true positives. Overall, the CNN method showed less confusion compared to the DNN and ANN methods. The confusion matrix of the LSTM on unseen subjects showed a very poor classification of *standing* and *ramp descent* activities, which were likely confused with *sitting* and *stair descent*, respectively. The *walking* activity was mildly confused with *ramp ascent* and *ramp descent* activities, while the *sitting* activity was not confused with any other activity. The unseen subject results for the CNN-LSTM hybrid showed an improved classification accuracy with only *standing* and *stair descent* being heavily confused with *sitting* and *ramp descent*, respectively. The validation results for unseen motion circuits from seen subjects showed that the lowest F1-score values were always assigned to *walking* and *stair descent* activities for all computational methods (see Table 3). The feature extraction process itself is unlikely to be the cause for this misinterpretation, since five different methods with five different feature extraction layers experienced the same problem. The most reasonable explanation for this phenomenon is that the sensor modalities failed to convey enough information that could be used to create more distinctive features. The confusion matrices with validation results in Figure 7c, Figure 8c, Figure 9c, Figure 10c and Figure 11c showed that the majority of *ramp ascent* false positives were predicted as *sitting*, indicating a mild confusion between the two classes for seen subjects. The validation matrices also showed that the majority of *walking* false positives were predicted as either *ramp ascent* or *ramp descent*, which was related to the minor discrepancies between walking on a ramp and normal walking.

## 6. Conclusions

In this work, a light-weight computational approach for the recognition of activities of daily living was presented. The proposed method used a systematic feature engineering procedure coupled with a single-layer artificial neural network. The analysis of the proposed light-weight ANN approach was performed using wearable data from the ENABL3S dataset. Furthermore, the proposed approach was compared against state-of-the-art deep learning methods such as DNNs, CNNs, LSTM and CNN-LSTM hybrid networks. The analysis showed that the proposed method was suitable for the recognition of ADLs with relatively small datasets and could generalize well to unseen motion circuits from seen users. The proposed ANN achieved a higher recognition accuracy than the benchmarking approaches for both seen and unseen data from seen subjects. The inference time for the proposed light-weight approach was at least thirty times less than any of the other deep learning approaches. A practical implementation would require more insight on sensor refresh rates and sensory channel synchronization in real time, which is an aspect that will be investigated in future work. Overall, the results from all experiments demonstrated that the light-weight ANN offered an alternative approach for a reliable and fast recognition of ADLs, making this method suitable for the development of portable robotic devices to assist subjects in real time.

## Figures and Tables

**Table 1:** Dataset subjects.

Subject	Age (Years)	Height (cm)	Weight (kg)
AB156	26	193	77
AB185	28	181	75
AB188	25	185	87
AB189	24	178	66
AB190	23	160	54
AB191	26	163	54
AB193	27	185	95
AB194	29	160	61

**Table 2:** Average training and validation accuracy results with seen subjects over eight folds.

Approach	Training Accuracy	Validation Accuracy
Light-weight ANN	98.81%	93.10%
DNN	97.92%	87.92%
CNN	97.55%	92.34%
LSTM	98.34%	92.99%
CNN-LSTM	97.75%	92.42%

**Table 3:** Average validation F1-score with seen subjects over eight folds (Highest F1-scores per row are presented in bold).

Approach	Sitting	Walking	R. Ascent	R. Descent	S. Ascent	S. Descent	Standing
Light-weight ANN	0.95	0.85	0.95	0.98	**0.996**	0.83	0.97
DNN	0.84	0.79	0.87	0.91	**0.92**	0.81	0.88
CNN	0.95	0.82	0.93	0.97	**0.995**	0.83	0.97
LSTM	0.95	0.84	0.95	0.97	**0.998**	0.81	0.98
CNN-LSTM	0.95	0.93	0.93	0.98	**0.99**	0.77	0.96

**Table 4:** Average training and validation accuracy results with seen subjects for the ANN using different sensor combinations.

Sensors	Training Accuracy	Validation Accuracy
IMUs only	99.43%	91.94%
Goniometers only	73.82%	71.51%
IMUs plus goniometers	98.81%	93.10%

**Table 5:** Average validation F1-score with seen subjects for the ANN using different sensor combinations (Highest F1-scores per row are presented in bold).

Sensors	Sitting	Walking	R. Ascent	R. Descent	S. Ascent	S. Descent	Standing
IMUs only	0.95	0.82	0.94	0.94	**0.995**	0.80	0.97
Goniometers only	0.88	0.51	0.69	0.64	**0.62**	0.72	0.82
IMUs plus goniometers	0.95	0.85	0.95	0.98	**0.996**	0.83	0.97

**Table 6:** Average testing accuracy with unseen subjects over eight folds.

Approach	Testing Accuracy
Light-weight ANN	84.77%
DNN	70.28%
CNN	86.78%
LSTM	86.9%
CNN-LSTM	86.36%

**Table 7:** Average testing F1-scores with unseen subjects over eight folds (Highest F1-scores per row are presented in bold).

Approach	Sitting	Walking	R. Ascent	R. Descent	S. Ascent	S. Descent	Standing
Light-weight ANN	0.89	0.76	0.86	0.82	**0.895**	0.83	0.87
DNN	**0.795**	0.63	0.68	0.62	0.71	0.68	0.78
CNN	0.90	0.79	0.88	0.83	**0.92**	0.87	0.88
LSTM	**0.898**	0.82	0.89	0.86	0.897	0.84	0.85
CNN-LSTM	0.87	0.84	**0.91**	0.84	0.89	0.83	0.85

**Table 8:** Average testing accuracy results with unseen subjects for the ANN using different sensor combinations.

Sensors	Testing Accuracy
IMUs only	79.55%
Goniometers only	69.96%
IMUs plus goniometers	84.77%

**Table 9:** Average testing F1-scores with unseen subjects for the ANN using different sensor combinations (Highest F1-scores per row are presented in bold).

Sensors	Sitting	Walking	R. Ascent	R. Descent	S. Ascent	S. Descent	Standing
IMUs only	**0.88**	0.65	0.77	0.77	0.80	0.83	0.84
Goniometers only	**0.84**	0.53	0.69	0.57	0.67	0.82	0.73
IMUs plus goniometers	0.89	0.76	0.86	0.82	**0.895**	0.83	0.87

## Data Availability

The study used an open-source dataset called ENABL3S which can be accessed at https://figshare.com/articles/dataset/Benchmark_datasets_for_bilateral_lower_limb_neuromechanical_signals_from_wearable_sensors_during_unassisted_locomotion_in_able-bodied_individuals/5362627 ( accessed on 28 March 2023).
